# Supplementary figures and images for: Evolution of Helicobacter spp: variability of virulence factors and their relationship to pathogenicity
Source: PeerJ. 2022 Aug 29;10:e13120. doi: 10.7717/peerj.13120 (PMC9435515; doi:10.7717/peerj.13120)

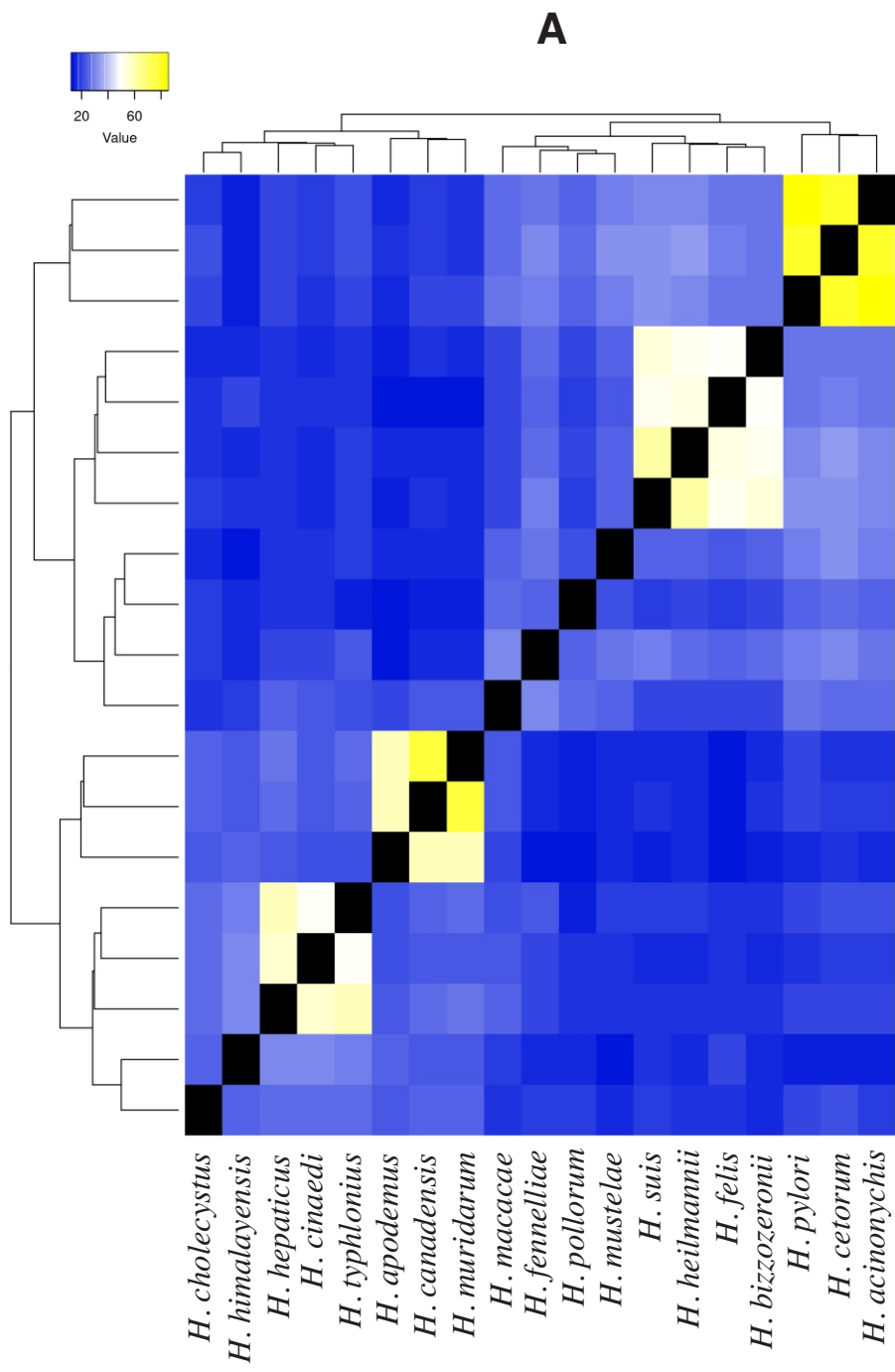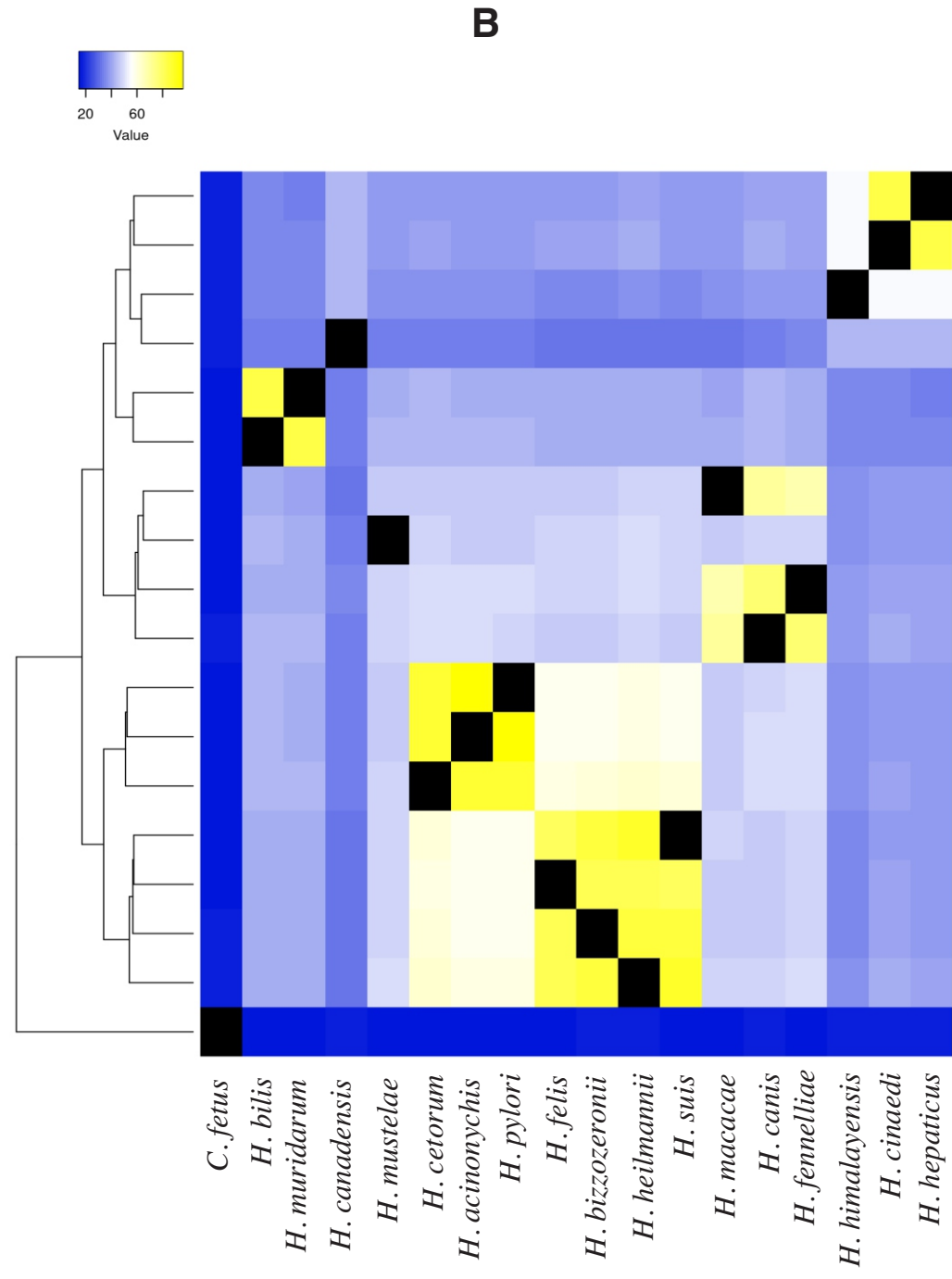

Supplement: Supplemental Information 1 — (A) Amino acid identity analyses for flgA gene in 19 Helicobacter species. (B)Amino acid identity analyses for flgE_2 gene in 18 Helicobacter species [file peerj-10-13120-s001.pdf]
